# Patent Foramen Ovale on Transthoracic Echocardiography and Brain White-Matter Hyperintensities: A Transportability Analysis and Practice-Anchored Risk Framework

**DOI:** 10.3390/jcm15124541

**Published:** 2026-06-11

**Authors:** Grigory Roytberg, Andrey Ardashev, Jeremiah Wasserlauf, Kevin Estrada, Evgeny Zhelyakov, Ariel Starr, Vyacheslav Koliev, Aleksey Amyaga, Evan Doubovikov, Arina Prokudina, Viktor Tcivkovskii, Janna Dorosh, Mikhail Nikogosyan, Yurii Karpenko, Igor R. Efimov, Natalia Kondratova, Daniil P. Aksenov

**Affiliations:** 1 Department of Cardiology, JSC “Medicine”, 125047 Moscow, Russia; contact@medicina.ru (G.R.); scdguidelines@gmail.com (E.Z.); s.koliev8@gmail.com (V.K.); 2 Department of Radiology, Endeavor Health Research Institute, Evanston, IL 60201, USA; kevin.estrada@endeavorhealth.org (K.E.); ariel.starr@endeavorhealth.org (A.S.); e.doubovikov@gmail.com (E.D.); 3 Department of Cardiology, Endeavor Health Cardiovascular Institute, Glenview, IL 60026, USA; jwasserlauf@northshore.org; 4 Department of Radiology, JSC “Medicine”, 125047 Moscow, Russia; fvzuf1975@gmail.com (A.A.); nikogosyan.mikhail@gmail.com (M.N.); kondratova@medicina.ru (N.K.); 5 Department of Internal Medicine, JSC “Medicine”, 125047 Moscow, Russia; arina674410@gmail.com (A.P.); jdorosh@medicina.ru (J.D.); 6 Department of Cardiovascular Surgery, JSC “Medicine”, 125047 Moscow, Russia; tsivkov@gmail.com; 7 Department of Internal Medicine and Cardiovascular Pathology, Odessa National Medical University, 65082 Odessa, Ukraine; arcard2@gmail.com; 8 Department of Cardiology, Northwestern University, Chicago, IL 60611, USA; igor.efimov@northwestern.edu; 9 Department of Biomedical Engineering, Northwestern University, Chicago, IL 60208, USA

**Keywords:** patent foramen ovale, white-matter hyperintensities, transthoracic echocardiography, cerebral injury, transportability, risk prediction, atrial cardiomyopathy, cardio-cerebral vulnerability, cardio-cerebral axis

## Abstract

**Background:** Meta-analyses suggest an association between patent foramen ovale (PFO) and white-matter hyperintensities (WMH), but pooled effect sizes do not clarify applicability to routine transthoracic echocardiography (TTE) or provide patient-level risk estimates. **Objective:** The objective of this study was to evaluate the association between TTE-detected PFO and MRI-defined WMH in routine practice and to develop a practice-anchored framework (PAMAP) that translates literature-derived evidence into individualized risk. **Methods**: We performed a retrospective, single-center, propensity-matched analysis of 149 adults undergoing TTE and brain MRI (37 PFO-positive, 112 controls). The primary endpoint was WMH (Fazekas ≥ 1). PAMAP synthesized 12 studies; 4 eligible studies were pooled using random-effects meta-analysis to derive a locked shunt coefficient (OR 3.65). The locked model used age and shunt (H); embolic context (E), and atrial stress (A) were neutral until refit. Transportability was assessed at the cohort level (expected vs. observed prevalence) and patient level (calibration, discrimination), followed by a minimal prespecified refit. **Results**: WMH was more frequent in PFO-positive versus control participants (54% vs. 32%). Literature-based expected prevalence approximated observed prevalence, supporting transportability. The locked model showed acceptable performance (calibration intercept 0.106; slope 0.912; Brier 0.188; AUC 0.756). A parsimonious refit improved performance (Brier 0.176; AUC 0.783), with the shunt term remaining significant (OR 2.45, 95% CI 1.23–4.88). **Conclusions**: PAMAP translates meta-analytic associations into a transportable patient-level risk framework. In routine TTE-defined PFO populations, the WMH association is preserved, suggesting that incidental PFO may mark early subclinical cerebral injury and enabling calibrated, individualized risk assessment.

## 1. Introduction

### 1.1. Background

Patent foramen ovale (PFO) persists in approximately one quarter of adults and has been variably associated with embolic stroke, migraine, decompression illness, and other cerebrovascular conditions [1,2,3]. Beyond overt infarction, multiple clinic- and population-based studies report a higher frequency of white-matter hyperintensities (WMH) in individuals with PFO. WMHs are clinically relevant, as they are associated with increased risks of stroke, cognitive decline, gait impairment, and mortality. However, reported effect sizes vary substantially across studies, reflecting differences in patient populations, diagnostic approaches, and clinical context. In particular, many studies rely on high-sensitivity detection techniques—such as contrast transesophageal echocardiography or transcranial Doppler—typically used in specialized research or stroke-evaluation settings rather than routine clinical practice. In addition, heterogeneous populations (e.g., general cohorts, migraine populations, divers) further complicate interpretation [1,2,3,4,5,6,7,8,9,10]. As a result, pooled estimates may combine fundamentally different phenotypes and may not directly inform risk in patients with PFO identified by routine transthoracic echocardiography (TTE).

Our institutional observations are consistent with prior reports, demonstrating a higher WMH burden in patients with incidental echocardiography-detected PFO and modest but consistent differences in atrial and diastolic echocardiographic indices compared with patients without PFO. These findings raise a critical translational question concerning whether literature-derived associations between PFO and cerebral white-matter injury are reproducible and clinically meaningful in routine practice and whether they can be translated into patient-level risk in TTE-defined PFO populations.

### 1.2. Study Rationale

Traditional meta-analyses are valuable for summarizing whether associations—such as that between PFO and white-matter lesions (WMLs)—are consistent across studies. However, they leave two critical gaps for clinical applicability. First, pooled effect estimates do not indicate whether literature-derived signals apply to individual clinical populations or provide calibrated patient-level risk at the bedside. Second, most single-center studies validate associations only within their own cohorts and rarely assess whether published effects remain stable when anchored to routine clinical phenotypes, such as PFO detected by standard TTE rather than research-grade shunt assessment.

These limitations are particularly relevant in the PFO–WML literature, which spans heterogeneous contexts, including population-based cohorts with near-null associations [1], clinic-based cohorts with stronger effects [4,5], phenotype-enriched populations [3,6], and mechanistic series that clarify lesion patterns but not prevalence [2,7,8]. As a result, current evidence synthesis may obscure clinically meaningful heterogeneity and provide limited guidance for risk interpretation in routine practice.

### 1.3. Objective

The objective of this study is to develop and test a practice-anchored, literature-trained predictive framework (PAMAP) that integrates routine TTE phenotyping with published evidence to generate transportable, patient-level risk estimates for PFO-associated WMH.

## 2. Methods

### 2.1. Study Overview

We conducted a retrospective, single-center observational study to evaluate the association between PFO detected on routine TTE and cerebral WMH identified on brain MRI. The study was designed to assess whether literature-derived associations between PFO and WMH are reproducible in routine clinical practice and to determine whether these signals can be translated into patient-level risk estimates.

To address this objective, we implemented a PAMAP framework that integrates evidence from published studies with real-world clinical data. The framework was structured to (i) align exposure definition with routine clinical phenotyping (TTE-detected PFO patients), (ii) incorporate literature-derived effect estimates as fixed model components, and (iii) evaluate transportability of these associations at both the cohort and patient levels prior to any local model updating.

All analyses were conducted using a predefined workflow, with subsequent steps including evidence assembly and tiering, model specification and locking, transportability testing, and minimal refit. This retrospective observational study was reviewed and approved by the Institutional Review Board of JSC “Medicine”. The requirement for informed consent was waived due to the retrospective nature of the study, which involved analysis of existing de-identified clinical and imaging data and posed minimal risk to participants.

### 2.2. Study Population

**Figure 1:**
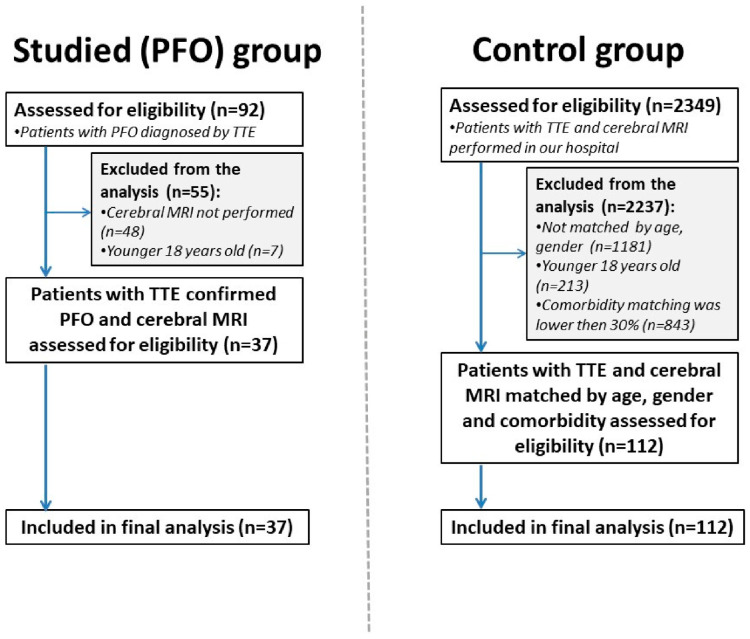
Patient selection flowchart. Abbreviations: MRI—magnetic resonance imaging, PFO—patent foramen ovale, TTE—transthoracic echocardiography.

We used data from a retrospective, single-center, case–control study that included patients admitted to our institution between January 2015 and December 2022 as the local transport cohort. A schematic of the patient selection process is shown in Figure 1, and detailed inclusion and exclusion criteria are summarized in Supplement Section S1. The PFO group consisted of consecutive patients aged 18–80 years with a PFO confirmed by transthoracic echocardiography. Patients with PFO who did not undergo brain magnetic resonance imaging (MRI) were excluded. The control group included consecutive patients without PFO who were evaluated or hospitalized during the same period, all of whom underwent both TTE and brain MRI. After applying the inclusion and exclusion criteria, the two cohorts were matched using propensity scores to reduce baseline differences. Demographic data, presenting symptoms, comorbidities, and medical histories were retrospectively reviewed and obtained from the institutional digital health records.

### 2.3. Outcomes, Definitions, and Statistical Analysis

The primary MRI endpoint for PAMAP transport analyses was any WMH, defined as Fazekas grade ≥ 1 on FLAIR. The Fazekas grading system classifies white-matter hyperintensities on MRI according to lesion extent and confluence, ranging from grade 0 (absence of lesions) to grade 3 (large confluent abnormalities involving extensive white-matter regions). In the present study, WMH positivity was defined as Fazekas grade ≥ 1. MRI interpretation was performed independently and blinded to echocardiographic findings, including PFO status, to reduce observational bias during WMH assessment.

Descriptive analyses used the matched cohort of 149 patients (112 controls and 37 PFO-positive patients). Continuous variables are presented as mean ± SD and were compared with Welch’s *t* test; categorical variables were compared with Fisher’s exact test. All *p* values are two-sided and should be interpreted descriptively given the retrospective matched design.

### 2.4. Imaging and Echocardiography Standards

MRI outcomes were defined according to STRIVE criteria and graded by the Fazekas scale. Echocardiographic parameters, including diastolic function indices, followed ASE recommendations. Detailed TTE and MRI acquisition protocols are provided in Supplement Sections S2 and S3. These harmonized standards were used to align the literature-trained model with the institutional validation cohort.

### 2.5. PAMAP Framework Overview

The Practice-Anchored, Literature-Trained, Mode-Aware Predictive (PAMAP) framework is a prespecified 4-step analytical approach designed to translate literature-derived associations into clinically interpretable, patient-level risk estimates. It aligns exposure definitions with routine clinical phenotyping (TTE-detected PFO), incorporates meta-analytic effect estimates as fixed model components, and evaluates transportability of these associations to real-world cohorts.

Step 1. Practice-Anchored Assembly and Tiering

The model defines the exposure as PFO detected on routine TTE—the phenotype cardiologists use in real-world care—rather than research-only shunt quantification. This aligns the analytical framework with the exposure used in our single-center dataset.

#### 2.5.1. Systematic Assembly and Tiering of PFO–MRI Literature

To establish a transparent, evidence-based foundation, we systematically collected and organized published studies examining the relationship between PFO and WMH/WMLs.

A structured search was performed using PubMed and Scopus databases (search window through March 2025: 1997–2025, reflecting the period from early mechanistic studies (Knauth et al., 1997 [3]) through modern quantitative MRI analyses (Niiyama et al., 2024 [10]) with combinations of the following terms: “patent foramen ovale” OR “right-to-left shunt” OR “atrial septal aneurysm,” AND “white-matter lesions” OR “leukoaraiosis” OR “white matter hyperintensities” OR “magnetic resonance imaging” OR “stroke” OR “migraine.” Inclusion required human studies reporting both PFO detection method (TTE, TEE, or TCD) and MRI brain findings (qualitative or quantitative). Abstract screening and full-text review were performed independently by two reviewers, with consensus adjudication.

Studies were screened and categorized into three evidence tiers according to methodological rigor and population characteristics:

- *Tier 1—Mechanistic or research-grade:* Controlled or mechanistic studies using contrast-enhanced transesophageal (TEE) or transcranial Doppler (TCD) shunt quantification and standardized MRI lesion scoring.
- *Tier 2—Clinical or hospital-based:* Consecutive symptomatic cohorts (e.g., stroke, migraine, or unexplained neurological symptoms) assessed by TEE/TTE and clinical MRI.
- *Tier 3—Population or low-intensity detection:* Community-based or registry cohorts, typically using non-contrast TTE with simplified WMH assessment.

For each study, key parameters were extracted: patient type, imaging modality, PFO detection method, and reported effect sizes (e.g., odds ratios or mean WMH burden differences). Effect sizes were standardized and aggregated by tier to describe the consistency and directionality of the PFO–WMH relationship across different study designs. This structured tiering enabled the integration of heterogeneous evidence into a unified analytical framework while making the results interpretable for clinicians.

Step 2. Development and Locking of the PAMAP Specification

#### 2.5.2. Literature-Trained and Locked Coefficient Building Based upon the Tiered Synthesis

Literature-anchored model—termed The PAMAP specification—was developed. The PAMAP framework was designed to represent mechanistically distinct but clinically recognizable pathways by which PFO may contribute to cerebral MRI abnormalities. Rather than developing a de novo local model. PAMAP coefficients were grounded in meta-analytic evidence, ensuring that estimated relationships reflected previously validated literature rather than single-center statistical fitting.

#### 2.5.3. Mode-Aware Risk Decomposition

PAMAP risk was partitioned into three interpretable modes that correspond to clinically meaningful mechanisms:

- *H-mode (Shunt/Hypoxemia):* Capturing right-to-left shunt burden, transient pressure gradients, and oxygen desaturation linked to PFO patency. To represent the shunt/hypoxemia (H) mode, we synthesized count-eligible studies [1,4,5,6] using a random-effects meta-analysis to derive a pooled odds ratio for the association between PFO and WMH/WMLs. This pooled estimate was then locked as the H-mode coefficient without further tuning before testing on the institutional cohort.
- *E-mode (Embolic Context):* Reflecting embolic potential from paradoxical embolism or coexisting venous thromboembolism traversing the PFO [2,11].
- *A-mode (Atrial/Diastolic Stress):* Encompassing atrial enlargement, diastolic dysfunction, or atrial cardiomyopathic remodeling that may coexist with or be potentiated by PFO physiology [9,10].

Each mode incorporated literature-derived variables (e.g., shunt size, migraine or stroke phenotype, left atrial strain, E/e′ ratio). By combining a structured evidence synthesis (Step 1) with a clinically interpretable, literature-anchored model (Step 2)**,** this approach bridges research findings with bedside decision support. Thus, it enables clinicians to understand PFO-related MRI findings through mechanistic and hemodynamic lenses—without requiring complex statistical retraining or purely data-driven inference. Model coefficients were locked before local validation to preserve external validity, ensuring that subsequent analyses tested the transportability of literature-based associations rather than overfitting to local data.

Step 3. Transportability Testing

After locking the PAMAP specification to published meta-analytic evidence, we evaluated how well the expected prevalence and directionality of PFO-related MRI findings translated to the institutional cohort. This step assessed average-level (group-wise) model transportability, meaning whether the general relationships observed in the literature could be reproduced in real-world patients before any local model adjustment. Two complementary tests were performed:

- *Cohort-level comparison:* Expected versus observed prevalence of white-matter hyperintensities (WMH; primary endpoint defined as Fazekas grade ≥ 1) was compared between our institutional cohort and the pooled reference value derived from the random-effects meta-analysis. Expected prevalence in the PFO-positive group was calculated from the control-group prevalence (p0) and the pooled odds ratio using p1 = (OR × p0)/(1 − p0 + OR × p0). Observed values were derived from the matched TTE-detected PFO cohort.
- *Subgroup-Level Concordance:* Differences in mean WMH burden and selected echocardiographic markers (e.g., left atrial size, E/e′ ratio, and PTFV1 when available) were evaluated between PFO-positive and PFO-negative groups.

We quantified the directional consistency (same versus opposite direction) and the magnitude ratio (observed effect ÷ expected effect) to determine the degree of alignment with the published evidence.

By doing so, we tested whether literature-derived trends—such as higher WMH burden in PFO-positive patients—remained evident when applied to a routine, TTE-based clinical population.

Step 4. Minimal Refit Procedure

Following average-level validation, individual-level translation was evaluated to test whether PAMAP could reliably predict MRI outcomes for individual patients:

- *Pre-Refit Evaluation:* The model’s predicted probability of WMH was compared against observed MRI findings. Calibration was quantified using the Brier score, calibration slope, and observed-to-expected ratio, while discrimination was assessed by the area under the receiver-operating characteristic curve (AUC).
- *Minimal refit and post-refit evaluation:* We first assessed calibration of the locked model using intercept, slope, Brier score, and AUC. We then performed a parsimonious refit that re-estimated only the pre-specified Age, H, E, and A terms; no new variables or interaction terms were introduced. Post-refit metrics were compared to determine whether limited local updating improved fit while preserving the literature-anchored structure.

### 2.6. Use of Generative Artificial Intelligence

Generative artificial intelligence (GenAI) tools were used exclusively to assist with language refinement, editorial organization of manuscript text, and preparation of the graphical abstract. No GenAI tools were used for study design, data collection, data curation, statistical analysis, neuroimaging interpretation, echocardiographic interpretation, model development, or generation of scientific conclusions. All scientific content, methodological decisions, data analyses, interpretations, and conclusions were independently developed, reviewed, and approved by the authors.

## 3. Results

### 3.1. Patients’ Baseline Clinical and Imaging Characteristics

We identified 92 patients in whom a PFO was documented on TTE. Among these, 48 did not undergo brain MRI and seven were younger than 18 years, leaving 37 patients with confirmed PFO and brain MRI available for analysis. For the control group, 2349 patients with both TTE and brain MRI were screened; after matching, 112 controls were retained. Demographic characteristics and traditional vascular risk factors were broadly comparable between groups. Symptoms such as headache, dizziness, weakness, and palpitations were numerically more frequent in the PFO group; among these, only dizziness remained clearly different on descriptive testing (Table 1).

#### 3.1.1. Transthoracic Echocardiography

Compared with controls, patients with PFO had higher LA anteroposterior diameter, LA volume, LAVI, RV area, PASP, and LVMI, together with a markedly higher prevalence of atrial septal aneurysm. Diastolic dysfunction grade distribution was similar between groups (Table 2). These findings support an overlapping PFO–atrial/diastolic phenotype without implying causality.

#### 3.1.2. PFO and Cerebral MRI

Brain MRI abnormalities were observed in 25 of 37 patients (68%) in the PFO group and 38 of 112 controls (34%) (*p* < 0.001). Any WMH (Fazekas ≥ 1) were present in 20 of 37 PFO-positive patients (54%) versus 36 of 112 controls (32%) (*p* = 0.020). Fazekas 1 accounted for most lesions; Fazekas 2 remained uncommon, and Fazekas 3 was not observed. Lacunes were infrequent and did not differ significantly between groups (3/37 vs. 2/112, *p* = 0.098). Moderate WMH burden (Fazekas 2) was numerically more frequent in the PFO group (11% vs. 6%) but remained uncommon overall (Table 3).

### 3.2. Literature Layer: Overview of All Evidence

#### 3.2.1. Study Identification and Evidence Structure

The literature assembly yielded 12 primary studies (Table 4 and Table 5) encompassing both quantitative and contextual evidence linking PFO with MRI-detected white-matter changes. Four studies provided count-eligible, quantitative data for meta-analysis, while eight additional studies contributed mechanistic or contextual insights relevant to specific modes of the PAMAP model (Hemodynamic/Shunt [H], Embolic [E], or Atrial/Diastolic [A]).

#### 3.2.2. Quantitative (Count-Eligible) Studies

The four pooled studies [1,4,5,6] collectively represented diverse populations—ranging from general cohorts (NOMAS) to migraine, pulmonary embolism, and high-altitude exposure settings. Most used contrast-enhanced echocardiography or TCD, ensuring consistent detection of right-to-left shunt. Across studies, the proportion of WMH or small brain infarct (SBI) in PFO-positive individuals ranged from 33% to 71%, compared with 5–28% among PFO-negative controls. Pooled meta-analytic estimates demonstrated a significant association between PFO and MRI-detected WMH:

- Fixed-effects pooled OR = 1.97 (95% CI 1.59–2.44)
- Random-effects pooled OR = 3.65 (95% CI 1.18–11.27)

Substantial heterogeneity was observed (Q = 32.95, df = 3; τ^2^ = 1.07; I^2^ = 90.9%), reflecting variability in population selection, imaging thresholds, and PFO detection modalities. These values provided the empirical basis for the shunt/hypoxemia (H-mode) coefficient in the PAMAP model.

The results showed a large, statistically significant overall effect (the odds of the event are higher in the intervention group) but with a very high degree of variability among the studies, where pooled fixed-effect OR = 1.97 (95% CI 1.59–2.44); pooled random-effects OR = 3.65 (95% CI 1.18–11.27); heterogeneity: Q = 32.95 (df = 3), τ^2^ = 1.066, I^2^ = 90.9%.

#### 3.2.3. Non-Quantitative/Contextual (Mechanistic) Studies

Eight additional studies contributed pattern-based or mechanistic context (Table 6):

- *E-mode (Embolic patterns):* Kim 2013 [2] and Steiner 1998 [11] demonstrated a higher prevalence of cortical and vertebrobasilar infarcts in PFO-related versus atrial fibrillation–related stroke, supporting paradoxical embolism mechanisms.
- *H-mode (Shunt–Hypoxemia):* Knauth 1997 [3] and Cabrera 2023 [12] emphasized lesion clustering in divers with large functional shunts, consistent with hypoxemic microbubble transit.
- *A-mode (Atrial/Diastolic):* Badea 2024 [9] and Niiyama 2024 [10] linked diastolic stress indices and left atrial metrics to higher periventricular WMH burden even in non-stroke cohorts.

Together, these studies contextualized the pooled quantitative signal, showing that the PFO–WMH link spans multiple, potentially overlapping biological pathways.

### 3.3. Model Layer: PAMAP Outputs Relative to Literature

The PAMAP model was initialized using literature-derived coefficients:

- *The shunt/hypoxemia (β_H)* coefficient was set equal to the pooled *random-effects* log-odds ratio (≈1.29).
- *The embolic (β_E) and atrial/diastolic (β_A)* components were retained with neutral priors (mean = 0; wide variance), subsequently informed qualitatively by contextual studies indicating embolic and atrial mechanisms.

This “locked” structure preserved fidelity to published evidence, ensuring that any subsequent calibration in local data represented *translational testing* rather than empirical re-estimation.

### 3.4. Cohort Layer: Institutional Patient Characterization

#### 3.4.1. Demographics and Baseline Characteristics

The matched analytical cohort comprised 149 adults (112 controls and 37 patients with TTE-detected PFO; approximately 73% female; mean age about 51 years). Baseline vascular risk factors were broadly comparable between groups. Compared with controls, the PFO group showed higher LAVI and PASP and a markedly higher prevalence of atrial septal aneurysm (Table 1 and Table 2).

#### 3.4.2. MRI Findings by PFO Status

WMH prevalence was higher among PFO-positive participants (20/37, 54%) than among PFO-negative controls (36/112, 32%) (Table 3). Fazekas 2 lesions were uncommon but numerically more frequent in the PFO group (4/37, 11% vs. 7/112, 6%). This pattern preserved the direction of the literature-derived association while showing attenuation relative to research-grade PFO ascertainment.

#### 3.4.3. Age-Stratified Trends

The PFO-related gradient in WMH prevalence was most pronounced in younger individuals (Table 3):

- Those <45 years old: 40.0% (PFO+) vs. 5.9% (PFO−)
- Those 45–60 years old: 46.7% vs. 28.3%
- Those >60 years old: 90.0% vs. 70.4%

This pattern suggests that, in younger patients, PFO may contribute to WMH independently of age-related small-vessel disease.

### 3.5. Fit of Our Data to the Literature-Trained Model (PAMAP)

#### 3.5.1. Average-Level Transport (Population Calibration)

**Figure 2:**
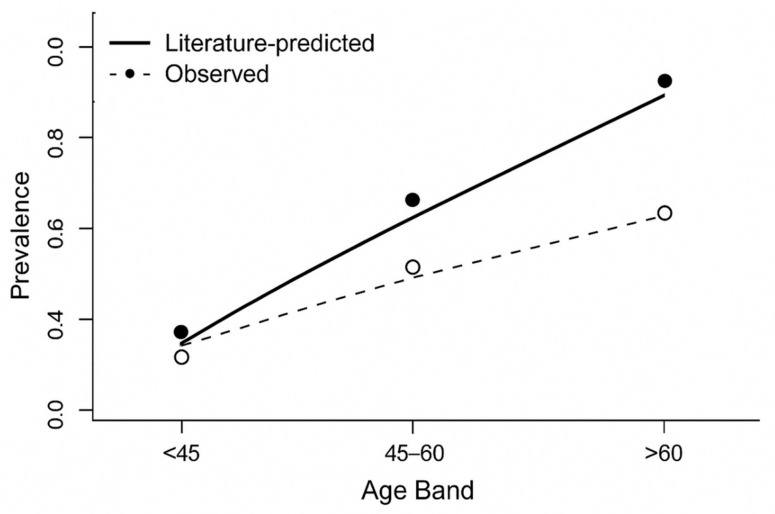
Expected vs. Observed Prevalence Across Age band.

At the cohort level, the literature-trained model preserved the direction of the published association when applied to routine TTE-defined PFO:

- Control prevalence (p0): approximately 0.32;
- Literature-predicted prevalence for PFO-positive patients (p1, model): approximately 0.63;
- Observed prevalence in the PFO-positive cohort (p1, observed): approximately 0.54; observed − expected = −0.09 (see Table 7, and Figure 2).

#### 3.5.2. Individual-Level Transport and Minimal Refit

Under the locked specification, the literature-derived shunt coefficient (β_H_locked = 1.293) was applied without local re-estimation. Model discrimination remained moderate (AUC 0.756) and calibration was acceptable (intercept 0.106; slope 0.912; Supplement Tables S2 and S3), supporting the feasibility of initial bedside translation from literature to routine-care data.

#### 3.5.3. Minimal Refit Phase

We then performed a parsimonious refit that re-estimated only the pre-specified Age, H, E, and A terms, without adding new variables or interactions. This limited update improved the Brier score from 0.188 to 0.176 and the AUC from 0.756 to 0.783 (Supplement Table S5). In the refit model, the shunt term remained significant (OR 2.452, 95% CI 1.231–4.881), whereas AF and the echo index were not statistically significant (Supplement Table S4). These post-refit estimates should be interpreted cautiously because of the modest sample size.

#### 3.5.4. Crosswalk to Literature

The minimally refit shunt coefficient (OR about 2.5) lies between the fixed-effect and random-effects literature estimates, suggesting that the routine-care cohort is directionally concordant with the published signal while less enriched than some mechanistic series. The approximately 9-percentage-point gap between expected and observed prevalence is consistent with attenuation from routine TTE detection rather than reversal of effect.

### 3.6. Clinical Relevance

In a routine-care setting, the literature-trained PAMAP framework generated interpretable, patient-level WMH risk estimates for TTE-detected PFO. Only limited local updating was required to improve fit, supporting the following practical inferences:

- PFO remains a measurable contributor to silent brain injury risk, even outside research-grade cohorts.
- Age and atrial stress exert additive but secondary effects.
- TTE-detected PFO, though less sensitive than TEE/TCD, still captures clinically meaningful brain–heart linkage.

Overall, these findings support the use of literature-trained, interpretable models such as PAMAP for translational quality benchmarking and individualized risk discussion while underscoring the need for external multi-center validation.

## 4. Discussion

Meta-analysis remains essential for establishing whether an association is present; however, it is inherently limited for clinical decision-making because it yields pooled effect estimates rather than calibrated probabilities at the patient level. In the context of PFO and WMH, this limitation is particularly relevant given the heterogeneity of populations, diagnostic approaches, and clinical phenotypes.

The PAMAP framework addresses this translational gap by using pooled evidence as an anchor while preserving mechanistic interpretability through its component domains (age, shunt, embolic, and atrial modes). Rather than replacing evidence synthesis, PAMAP extends it by testing whether literature-derived signals transport to routine TTE-defined PFO and by converting pooled associations into calibrated, patient-level risk estimates.

In this study, the literature-derived shunt signal remained applicable at both the cohort and individual levels, supporting the premise that heterogeneous evidence can be translated into clinically meaningful predictions when appropriately structured. Importantly, the persistence of the shunt/hypoxemia component after minimal refit suggests that right-to-left shunting represents a stable and transferable determinant of WMH risk, even in routine clinical phenotypes defined by standard TTE. Clinically, at the bedside, PFO should be interpreted using this structured approach, incorporating (i) shunt evidence, (ii) embolic plausibility, and (iii) atrial/diastolic dysfunction features. PAMAP, as an instrument, operationalizes this by combining H–E–A domains into a unified probability estimate, enabling transition from PFO presence to quantified, patient-specific WMH risk. Its applicability, not re-estimation alone, is key to converting meta-analytic signals into actionable clinical tools.

### 4.1. Pathophysiological Context

Clinical symptoms were nonspecific in both groups and should be interpreted cautiously, given the retrospective, descriptive design. In contrast, the most reproducible signal in this study was the imaging phenotype itself—WMH—which represents cumulative cerebral injury related to small-vessel disease, embolic phenomena, and impaired perfusion, and is associated with subsequent stroke, cognitive decline, and functional impairment [14].

In the setting of PFO, several interacting mechanisms may contribute to this phenotype. Right-to-left shunting provides a direct pathway for paradoxical embolism or microembolization into the cerebral circulation. In parallel, shunt-related hypoxemia may impair cerebrovascular autoregulation and reduce perfusion reserve, further predisposing to white-matter injury.

Importantly, the observed differences in atrial size and diastolic indices suggest an accompanying atrial–hemodynamic substrate consistent with early atrial cardiomyopathy [15]. Such a substrate may promote blood stasis within the left atrium, increasing susceptibility to embolic phenomena even in the absence of clinically overt atrial fibrillation.

Taken together, these findings support a multi-component model in which shunt physiology, atrial remodeling, and cerebral perfusion abnormalities converge to produce the WMH phenotype. This integrated framework is consistent with the structure of the PAMAP model and provides biological plausibility for the observed transportability of the PFO–WMH association in routine clinical practice.

### 4.2. Cardiac Structural and Echocardiographic Correlates

Although PFO in this study was identified using routine TTE, its association with WMH suggests that even hemodynamically mild forms may be accompanied by intermittent interatrial flow and subtle abnormalities in diastolic function. These findings point to a potential overlap between PFO and an underlying atrial–hemodynamic phenotype.

Compared with controls, patients with PFO demonstrated higher left atrial and left ventricular indices, including LAD, LAVI, LVMI, and pulmonary artery pressure, although values remained within reference ranges. The consistent directionality of these differences supports the presence of early atrial remodeling.

Taken together, these observations are consistent with an emerging model in which PFO interacts with an atrial cardiomyopathy, rather than functioning solely as an isolated anatomical conduit [15]. This interpretation aligns with the broader concept of a cardio-cerebral axis linking atrial dysfunction, shunt physiology, and cerebral white-matter injury.

### 4.3. Modeling Framework and Transportability

A central challenge in clinical translation is determining whether associations derived from published studies apply to local patient populations and can inform individual risk. Meta-analyses provide pooled effect estimates across studies, whereas single-center analyses reflect local experience; however, these approaches operate in parallel and do not directly address whether literature-derived signals are transferable to routine clinical practice.

To bridge this gap, we adopted a literature-trained approach in which the effect size linking PFO to WMH was derived from prior studies and incorporated as a fixed (“locked”) coefficient. By design, this coefficient was not re-estimated using local data, allowing for direct testing of whether the published association remains valid in a real-world TTE-defined cohort.

This framework enables two complementary and clinically interpretable transportability assessments. First, average-level calibration compares the expected WMH prevalence—derived from the locked effect size—with the observed prevalence in the local cohort. Second, patient-level calibration evaluates agreement between predicted and observed outcomes using standard performance metrics, including calibration intercept and slope, Brier score, and the area under the receiver operating characteristic curve (AUC). Together, these steps provide a structured approach to determine whether literature-derived associations can be translated into clinically meaningful, patient-level risk estimates.

### 4.4. Clinical Interpretation

Our findings indicate that patients seen in TTE routine with PFO often already show MRI evidence of subclinical cerebral injury. In this context, the most consistent and reproducible signal was the WMH phenotype itself, rather than any specific symptom profile (but dizziness), emphasizing the importance of imaging-defined disease burden in individual risk assessment.

The PAMAP framework provides several clinically relevant advantages. First, it enables patient-level risk estimation by integrating routinely available variables, including age, PFO status on TTE, and echocardiographic indices. Second, the model preserves physiological interpretability by partitioning risk into shunt-related, embolic, and atrial components, thereby linking prediction to underlying mechanisms rather than treating risk as a purely statistical construct.

Third, the framework demonstrated applicability within a routine-care cohort, with close agreement between expected and observed WMH prevalence and acceptable calibration, suggesting that literature-derived associations can be meaningfully applied in real-world settings. At the same time, the need for external validation remains, particularly across diverse populations and imaging practices.

Finally, PAMAP is aligned with the phenotype clinicians actually encounter—PFO detected incidentally on standard TTE—thereby avoiding the selection biases inherent to research cohorts defined by TEE or TCD. In this way, PAMAP complements rather than replaces meta-analysis: whereas meta-analysis defines the existence of an association, this framework operationalizes that evidence into a clinically interpretable and transportable estimate of individual risk.

### 4.5. Clinical Implications

The central clinical question was whether the published PFO–WMH association retains relevance in routine cardiology practice. The PAMAP framework addresses this by combining baseline WMH prevalence with a small set of clinically accessible variables—age, PFO status on TTE, atrial rhythm context, and echocardiographic indices—to generate absolute risk estimates at the individual level.

This approach enables two complementary use cases. In settings without local outcome data, the locked model provides a direct translation of published evidence into bedside risk estimation. In settings with local data, limited recalibration using the same prespecified variables allows for refinement without departing from the original evidence structure.

By design, this framework shifts the role of meta-analysis from a terminal summary measure to an applicable component of possible clinical prediction. The locked model tests whether the literature signal survives in routine care, whereas the minimal refit quantifies the degree of local adaptation required. Because the variable set is fixed and mechanistically interpretable, the model can be implemented across a variety of clinical environments without sacrificing consistency with the underlying evidence base.

Future studies should evaluate whether mechanism-based phenotyping using the H–E–A framework may help identify patient subgroups with differing biological pathways or phenotypes of cerebrovascular vulnerability. In particular, prospective investigations are needed to determine whether patients characterized by prominent shunt physiology, embolic susceptibility, or atrial–hemodynamic substrate demonstrate differential risk trajectories or derive benefit from intensified surveillance, rhythm monitoring, medical therapy, or potentially PFO closure. However, the present findings are observational and hypothesis-generating and should not be interpreted as evidence supporting therapeutic intervention or closure strategies at this stage.

### 4.6. Study Limitations

1. This was a retrospective, single-center study, and PFO classification was based on routine transthoracic echocardiography color Doppler rather than on contrast-enhanced transesophageal echocardiography or transcranial Doppler, which may have led to underdetection of small or transient shunts. The relatively small number of PFO-positive patients limited statistical precision and increased the risk of overinterpretation, particularly for subgroup analyses, patient-level calibration, and the parsimonious refit. Accordingly, the present study should be interpreted as an exploratory, proof-of-concept transportability analysis rather than a definitive predictive modeling study.

2. Selection bias also represents an important limitation. Nearly half of initially identified PFO-positive patients did not undergo brain MRI and were therefore excluded, potentially enriching the analyzed cohort for individuals with neurological symptoms or greater clinical concern. Consequently, the observed WMH prevalence may not reflect the broader population of patients with incidentally detected PFO in routine clinical practice.

3. White matter hyperintensities were assessed using visual Fazekas grading rather than quantitative volumetric measures. In addition, the literature-derived shunt coefficient was based on a limited number (just 4) of currently available heterogeneous studies spanning different populations, imaging protocols, and ascertainment strategies, resulting in substantial statistical heterogeneity. Therefore, the pooled estimate should be interpreted as an approximate transportability anchor rather than a universally stable biological effect size.

4. The present analysis represents an internal assessment of transportability within a single clinical setting rather than formal external validation across independent cohorts**. Despite propensity matching, residual confounding remains plausible, particularly from vascular risk burden, referral patterns, shunt magnitude, subclinical atrial cardiomyopathy, and occult atrial fibrillation, which may influence both PFO status and WMH burden. Consequently, although the H–E–A framework is mechanistically informed and biologically grounded, the present observational analysis does not establish causality. Rather, the framework is intended to provide a clinically interpretable structure for contextualizing PFO-associated cerebral vulnerability and translating literature-derived associations into patient-level risk estimates.

5. A younger-age sensitivity analysis was considered because younger patients may represent a phenotype less confounded by age-related vascular disease and therefore more reflective of shunt-related mechanisms. However, the limited number of PFO-positive patients within age-stratified subgroups substantially reduced statistical stability and interpretability. Future multicenter studies with larger cohorts should specifically evaluate younger populations to better define the mechanistic contribution of PFO-associated shunting to WMH burden.

The limitations above underscore the need for prospective, multicenter studies with standardized imaging protocols, quantitative WMH assessment, and broader phenotypic and age characterizations to confirm the generalizability and clinical utility of the framework.

## 5. Conclusions

In a routine TTE-defined population, literature-derived associations between PFO and WMH demonstrated measurable transportability at both the cohort and patient levels, supporting the feasibility of translating heterogeneous evidence into clinically interpretable risk estimates. Rather than functioning as a definitive predictive model, the PAMAP framework should be viewed as an exploratory, mechanistically informed approach for contextualizing patient-level cerebrovascular vulnerability through integration of shunt physiology, embolic context, and atrial–hemodynamic substrate.

These findings suggest that PFO detected on routine transthoracic echocardiography may represent not only an anatomical variant but also a potential marker of subclinical cardio-cerebral vulnerability in selected clinical contexts. Future prospective multicenter studies are warranted to validate the framework, evaluate its performance across diverse populations, and determine whether H–E–A phenotyping may ultimately contribute to mechanism-directed surveillance or therapeutic strategies, including potential patient selection for intensified monitoring or future PFO-targeted interventions.

## Figures and Tables

**Table 1:** Demographics and clinical features of the patients.

	No PFO (*n* = 112)	PFO (*n* = 37)	*p* Value
Sex, male/female	32/80	9/28	0.676
Age, years	51 ± 14.8	51.49 ± 12.9	0.848
Body mass index, kg/m^2^	24.95 ± 3.00	24.50 ± 3.25	0.460
Smoking	9 (8%)	4 (11%)	0.737
Symptoms			
headache	67 (60%)	25 (68%)	0.441
dizziness	32 (29%)	19 (51%)	0.016
weakness	52 (46%)	23 (62%)	0.129
palpitation	31 (28%)	16 (43%)	0.102
Comorbidities			
Arterial hypertension	63 (56%)	23 (62%)	0.569
Coronary artery disease	7 (6%)	2 (5%)	1.000
Chronic kidney disease	1 (1%)	3 (8%)	0.047
Anemia	3 (3%)	2 (5%)	0.598
Carotid atherosclerosis	40 (36%)	18 (49%)	0.178
Prior stroke	2 (2%)	1 (3%)	1.000
Prior TIA	2 (2%)	1 (3%)	1.000
Migraine	11 (10%)	5 (14%)	0.546
Diabetes mellitus	12 (11%)	3 (8%)	0.763
Atrial fibrillation or atrial flutter	4 (4%)	1 (3%)	1.000
Supraventricular tachycardia	9 (8%)	3 (8%)	1.000
ECG			
PR, ms	158.19 ± 27.47	153.45 ± 23.7	0.315
QRS, ms	89.1 ± 18.9	93.4 ± 11.6	0.103
Right bundle branch block	11 (10%)	3 (8%)	1.000
Left bundle branch block	1 (1%)	0	1.000

**Table 2:** Comparison of TTE characteristics between groups.

	No PFO (*n* = 112)	PFO (*n* = 37)	*p* Value
LA anteroposterior diameter, mm	34.5 ± 4.2	37.0 ± 4.3	0.003
LA volume, ml	42.0 ± 4.5	45.0 ± 3.0	<0.001
LAVI, mL/m^2^	23.7 ± 4.5	26.8 ± 5.1	0.002
RA area, cm^2^	19.5 ± 6.2	15.6 ± 2.8	<0.001
RV area, cm^2^	26.7 ± 3.9	28.9 ± 3.8	0.004
PASP, mmHg	24.4 ± 4.8	27.1 ± 4.1	0.001
Left ventricle ejection fraction, %	65.3 ± 2.3	67.2 ± 4.3	0.012
LVMI, g/m^2^	70.2 ± 20.1	84.7 ± 17.8	<0.001
Diastolic dysfunction	33 (29%)	11 (30%)	1.000
Grade 1	31 (28%)	10 (27%)	1.000
Grade 2	-	-	-
Grade 3	2 (2%)	1 (3%)	1.000
Atrial septal aneurysm	2 (2%)	10 (27%)	<0.001

Abbreviations: LA—left atrium; LAVI—left atrial volume index; LVMI—left ventricular mass index; PASP—pulmonary artery systolic pressure; RA—right atrium; RV—right ventricle.

**Table 3:** Comparison of cerebral MRI characteristics and age-stratified WMH prevalence by PFO status.

	No PFO (*n* = 112)	PFO (*n* = 37)	*p* Value
Any neuroimaging abnormality	38 (34%)	25 (68%)	<0.001
Lacunes	2 (2%)	3 (8%)	0.098
WMH (Fazekas ≥ 1)	36 (32%)	20 (54%)	0.020
Fazekas 1	29 (26%)	16 (43%)	0.063
Fazekas 2	7 (6%)	4 (11%)	0.467
Age-stratified WMH prevalence			*p* value
<45	2/34 (5.9%)	4/10 (40.0%)	0.018
45–60	13/46 (28.3%)	7/15 (46.7%)	0.216
>60	19/27 (70.4%)	9/10 (90.0%)	0.393

**Table 4:** Count-eligible studies used for pooling.

Study (Year)	Tier	Population/Setting	PFO Detection	Outcome Measure	wml_pfo_n	WML_pfo_N	WML_ctrl_n	wml_ctrl_N	TEE_or_Contrast	Notes/Mechanistic Context
Di Tullio 2013, JACC [1]	3	population (NOMAS) MRI subcohort	contrast_TTE + Valsalva	SBI; WMH-Q4	10.0	60.0	42.0	300.0	1.0	Table 1 (counts), Table 4 (aORs); KM Figure 1A/B.
Clergeau 2009_Stroke [4]	2	acute PE cohort	contrast_TTE + Valsalva	SBI on DWI	5.0	15.0	1.0	45.0	1.0	OR 34.9 (3.1–394.3); DWI + FLAIR 1.5T; blinded.
Liu 2021_BMC [5]	2	high-altitude clinic 100 vs. 100	contrast_echo + diameter	ischemic lesions (3T FLAIR/DWI)	71.0	100.0	19.0	100.0	1.0	>4 mm 37/39; 2–4 mm 24/35; <2 mm 10/26 (trend *p* < 0.001).
Yeo 2022_FrontNeurol [6]	2	migraine meta-analysis (8 studies)	contrast_TCD/echo	WMH presence (meta)	338.0	576.0	256.0	549.0	1.0	Pooled OR 1.56 (1.05–2.34); adjusted pooled 3.84 (2.05–7.19).

Notes: The table summarizes representative studies across tiers, showing population type, PFO detection method, MRI outcome, effects, and context. Findings in the Notes column are summarized from the cited source publications. Any table, figure, or page references in the Notes column refer to the cited source publication, not to the present manuscript. For clinicians, the table highlights several key insights: (1) *Detection method matters:* Tier 1 studies (mechanistic approach) using TEE or contrast-TCD often report stronger associations than Tier 3 (population) studies with routine TTE. This emphasizes that under-ascertainment can dilute observed effects, showing near-null results likely due to the limited sensitivity of TTE in routine clinical practice. (2) *Population selection influences findings* (Tier 2): Symptomatic or enriched cohorts (stroke, migraine, divers) tend to show more robust WMH associations, while general population cohorts may show minimal effects. (3) *Mechanistic implications are visible* (Tier 1): The table links PFO presence with plausible biological mechanisms, such as embolic load or hypoxemia, making the associations more clinically plausible. Abbreviations: DWI—diffusion-weighted imaging, FLAIR—fluid-attenuated inversion recovery, OR—odds ratio, MRI—magnetic resonance imaging, PFO—patent foramen ovale, TCD—transcranial Doppler, TEE—transesophageal echocardiography, TTE—transthoracic echocardiography, WMH—white-matter hyperintensities, WML—white-matter lesions.

**Table 5:** Per-study 2 × 2 data and log-odds ratios.

study_id	a	b	c	D	log_or	var
DiTullio 2013_JACC [1]	10	50	42	258	0.206	0.148
Clergeau 2009_Stroke [4]	5	10	1	44	3.091	1.323
Liu 2021_BMC [5]	71	29	19	81	2.345	0.114
Yeo 2022_FrontNeurol [6]	338	238	256	293	0.486	0.014

**Table 6:** Mechanistic/pattern evidence from cited source publications (not pooled).

Study (Year)	Tier	Population/Setting	PFO Detection	Outcome Measure	Notes *	Mechanistic Mode (Used)
Kim 2013_Stroke [2]	1	cryptogenic PFO-stroke vs. AF-stroke	TEE + contrast; ASA	lesion pattern; angiography	Single cortical 34.2% vs. 3.1%; multiple small 23.1% vs. 5.9%; VB 44.4% vs. 22.9%; no-occlusion 65.8% vs. 28.5% (Tables 1 and 2; Figures 2 and 3).	E (embolic patterns)
Knauth 1997_BMJ [3]	1	sport divers	contrast_TCD + Valsalva; >20 bubbles	multiple lesions prevalence	Multiple lesions only with large PFO (*p* = 0.004); text pp. 702–703; FLAIR protocol.	H (shunt/RLS context)
Badea 2024_FrontNeurol [9]	-	TEE-confirmed PFO; stroke/TIA vs. no-stroke	TEE + c-TCD (Spencer)	WMH volume (Quantib ND)	WMH vol excl stroke: 0.27 vs. 0.08 cm^3^ (*p* = 0.019); ICC = 0.98; Quade ANCOVA.	A (atrial/diastolic)
Niiyama 2024_JSCVD [10]	3	multicenter cryptogenic registry	TEE bubbles; ASA	PVH/DSWMH (Fazekas); recurrence	Groups No-RLS 179/RLS 90/HR-PFO 60 (Figure 1); PVH HR 3.369 (1.103–10.294) (Table 4).	A (atrial/diastolic)
Wu 2022_JTT [8]	-	pattern taxonomy	TEE (PFO); CTA/MRA (aCSVD); MRBTI ± CE-MRV (CVT)	WML pattern and burden; PSM	Figures 3–5 (patterns); Table 3 (PSM burden).	Context/Pattern
Severa 2021_JNS [7]	1	MS 13 vs. PFO-WML 13		WML morphology/topography	Pattern discrimination features; Abstract; Methods/Results.	Context/Pattern
Cabrera 2023_JCM [12]	-	asymptomatic divers vs. controls	contrast_TTE (bubble-graded)	cWML; ASL perfusion	PFO 10/38 (26.3%); cWML 4/38 (10.5%); no PFO–cWML association (*p* ≈ 0.95).	H (shunt/RLS context)
Steiner 1998_Stroke [11]	-	ischemic stroke cohort (TEE subset)	TEE + contrast; size mm	acute infarct pattern	Med/large PFO vs. small/none: superficial 50% vs. 21%; VB 64% vs. 33%; *p* ≈ 0.02–0.05.	E (embolic patterns)
Cao 2022_BrainSci [13]	-	narrative review (migraine and PFO)		WMH evidence; mechanisms	Posterior WMH; detection hierarchy; E/H mechanisms.	Context/Pattern

Abbreviations: aCSVD—atherosclerotic cerebral small vessel disease, AF—atrial fibrillation, ASA—atrial septal aneurism, CE-MRV—contrast-enhanced magnetic resonance venography, CTA—computed tomography angiography, CVT—cerebral venous thrombosis, DSWMH—deep and subcortical white matter hyperintensity, HR—hazard ratio, MRA—magnetic resonance angiography, MRBTI—magnetic resonance black blood thrombus imaging, PFO—patent foramen ovale, PSM—propensity score matching, PVH—periventricular hyperintensity, RLS—right-to-left shunt, TCD—transcranial Doppler, TEE—transesophageal echocardiography, TTE—transthoracic echocardiography, TIA—transient ischemic attack, WMH—white-matter hyperintensities, WML—white-matter lesions. * Note: Findings in the Notes column are summarized from the cited source publications. Any table, figure, or page references in the Notes column refer to the cited source publication, not to the present manuscript.

**Table 7:** Average-level transport: expected versus observed WMH prevalence.

Quantity	Definition/Formula	Value	Source
Control prevalence (p_0_)	Mean (WMH|PFO−)	0.32	Our cohort
Expected PFO+ prevalence (p_1_, model)	(3.65 × p0)/(1 − p0 + 3.65 × p0)	0.63	Literature OR_RE
Observed PFO+ prevalence (p_1_)	Mean (WMH|PFO+)	0.54	Our cohort
Observed − expected	-	−0.09	-

Interpretation: The observed WMH prevalence in the TTE-detected PFO cohort remained directionally consistent with the meta-analytic expectation, although attenuated relative to the pooled estimate. This pattern is compatible with routine TTE under-ascertainment and the less enriched nature of the clinical cohort.

## Data Availability

De-identified patient-level data, the study-level literature extraction table, and a reviewer-accessible executable code bundle (Supplement Section S6) are provided as Supplementary Materials. The current manuscript corresponds to the parsimonious Age/H(shunt)/E(embolic)/A(atrial) refit workflow implemented in the accompanying code package.

## Supplementary Materials

The following supporting information can be downloaded at: https://www.mdpi.com/article/10.3390/jcm15124541/s1, Supplement Section S1: Table S1: Study inclusion and exclusion criteria; Supplement Section S2: Transthoracic Echocardiography (TTE); Supplement Section S3: Figure S1: Lacune: Ovoid fluid-filled cavity; Figure S2: Variants of white matter changes: Fazekas 0: Normal, Fazekas 1: Multiple punctate lesions, Fazekas 2: Beginning confluences of lesions (bridging), Fazekas 3: Large confluent lesions, Supplement Section S4: Table S2: Locked parameters used; Table S3: Model performance under locked priors; Supplement Section S5: Table S4: Minimal Refit Coefficients; Table S5: Fit metrics before and after minimal refit; Supplement Section S6: PAMAP Supplementary Code Package. References [16,17,18,19] are cited in the supplementary materials.
