# Supplementary material for: Patent Foramen Ovale on Transthoracic Echocardiography and Brain White-Matter Hyperintensities: A Transportability Analysis and Practice-Anchored Risk Framework
Source: J Clin Med. 2026 Jun 11;15(12):4541. doi: 10.3390/jcm15124541 (PMC13301136; doi:10.3390/jcm15124541)
Supplement: Supplementary file 1 [file jcm-15-04541-s001.zip › jcm-4322739-supplementary/jcm-4322739-supplementary.pdf]

Contents

(Section S1) Inclusion and exclusion criteria. 2

Table S1. Study inclusion and exclusion criteria 2

(Section S2) Transthoracic Echocardiography (TTE). 3

(Section S3) Cerebral Magnetic Resonance Imaging (MRI). 4

(Section S4) Individual-level transport and minimal refit. 7

Table S2. Locked parameters used 7

Table S3. Model performance under locked priors 8

(Section S5) Minimal refit phase. 9

Table S4. Minimal Refit Coefficients (OR, 95 % CI) 9

Table S5. Fit metrics before and after minimal refit 9

(Section S6) Reproducibility resources and executable code 10

References 11

(Section S1) Inclusion and exclusion criteria.

Table S1. Study inclusion and exclusion criteria

**Study group:**
**Inclusion criteria:**
1. Age ≥ 18 years
2. Diagnosis of PFO according to TTE reports
3. Cerebral MRI study
**Exclusion criteria:**
1. Age <18 years
2. Absence of cerebral MRI study
**Control group:**
**Inclusion criteria:**
1. Age ≥ 18 years
2. Cerebral MRI study
3. TTE without mention of PFO, ASD, ASA in reports
**Exclusion criteria:**
1. Age <18 years
2. Age ≥ 80 years
3. NO Cerebral MRI study
Abbreviations: MRI: magnetic resonance imaging; PFO: patent foramen ovale; TIA: transient ischemic attack.

(Section S2) Transthoracic Echocardiography (TTE).

TTE parameters were obtained by retrospective review of echocardiography reports. All examinations were performed during ambulatory visits using standard imaging planes in accordance with the recommendations of the American Society of Echocardiography.

The following parameters were recorded: left atrial (LA) anteroposterior diameter, LA volume, LA volume index (LAVi), right atrial (RA) area, left ventricular end-diastolic diameter (LVEDD), left ventricular end-systolic diameter (LVESD), left ventricular ejection fraction (LVEF), right ventricular (RV) area, left ventricular mass index (LVMI), and pulmonary artery systolic pressure (PASP).

Left ventricular diastolic function was graded according to established criteria [1]:

Grade 1 (impaired relaxation): E/A ratio <0.8, deceleration time >200–240 ms, and E/e′ ratio ≤8;

Grade 2 (pseudonormal pattern): E/A ratio 0.8–1.5, deceleration time 150–200 ms, and E/e′ ratio 9–12;

Grade 3 (restrictive filling): E/A ratio ≥2 and deceleration time <150 ms.

The presence and severity of mitral, tricuspid, and aortic regurgitation were documented. Color flow Doppler imaging was used to assess the presence of right-to-left (R→L) or left-to-right (L→R) shunting across the foramen ovale.

An *atrial septal aneurysm (ASA)* was diagnosed when the interatrial septum demonstrated a ≥10 mm midline excursion in M-mode, or a total bidirectional excursion >15 mm [2, 3].

(Section S3) Cerebral Magnetic Resonance Imaging (MRI).

All patients in the PFO and control groups underwent brain MRI using a Siemens Skyra 3T scanner equipped with a 48-channel head coil. The standardized imaging protocol included 3D T1-weighted gradient echo, 3D T2-weighted turbo spin echo, 3D T2*-weighted gradient echo or susceptibility-weighted imaging (SWI), 3D fluid-attenuated inversion recovery (FLAIR), and diffusion-weighted imaging (DWI). Detailed Scan parameters for the Siemens Skyra 3T MRI (48-channel head coil) were:

DWI repetition time / echo time (TR/TE) = 7400/73 milliseconds (ms); field of view (FOV) = 240x240 mm; matrix = 160x160; b values of 0 and 1000 s/mm2 ; thickness = 3 mm, intersection gap = 0.9 mm; NEX 1.

T1 TR/TE = 1600/2.8 ms; FOV = 249x249 mm; matrix = 256x248; thickness = 1mm; intersection gap = 0; NEX 1.

T2 TR/TE = 5100/89 ms; FOV = 185x220 mm; matrix = 640x324; thickness = 5mm; intersection gap = 1; NEX 3.

FLAIR TR/TE = 9000/97 ms; FOV = 199x220 mm; matrix = 384x348; thickness = 5mm; intersection gap = 1; NEX 1.

SWI TR/TE = 5100/89 ms; FOV = 185x220 mm; matrix = 640x324; thickness = 5mm; intersection gap = 1; NEX 3.

3D-TOF TR/TE = 5100/89 ms; FOV = 185x220 mm; matrix = 640x324; thickness = 5mm; intersection gap = 1; NEX 3.

All imaging data were retrospectively collected and independently reviewed by three experienced neuroradiologists, who were blinded to the group assignment. Image review and annotation were performed using RadiAnt DICOM Viewer (version 2025.1). Extracted data were tabulated for analysis in Microsoft Excel (version 2016, Microsoft Corporation, Seattle, USA).

Neuroimaging findings assessed included *recent subcortical infarcts, lacunes, white matter hyperintensities (WMH),* and *cerebral microbleeds.* Imaging features were defined and classified in accordance with the STandards for ReportIng Vascular changes on nEuroimaging (STRIVE) criteria and the Fazekas scale [4].

For PAMAP transport analyses, the primary neuroimaging endpoint was any WMH, defined as Fazekas grade ≥1 on FLAIR. Descriptive MRI summaries additionally report Fazekas subcategories and other neuroimaging abnormalities.

Definitions and Imaging Characteristics

Recent subcortical infarcts: Appeared slightly hypointense on T1-weighted, hyperintense on T2/FLAIR, and demonstrated restricted diffusion on DWI sequences.

Lacunes: Defined as round or ovoid, subcortical, fluid-filled cavities (signal similar to cerebrospinal fluid) measuring 3–15 mm in diameter, typically located within the territory of a single perforating arteriole. On FLAIR images, lacunes usually exhibit a central CSF-like hypointensity with a peripheral hyperintense rim, though the rim may be absent. Occasionally, a hyperintense rim may also surround enlarged perivascular spaces when they traverse areas of WMH. In some cases, incomplete CSF suppression on FLAIR may cause the entire lesion to appear hyperintense, despite a CSF-like signal on T1- and T2-weighted sequences (**Figure S1**).

White matter lesions (WMLs) / white matter hyperintensities (WMH): Defined as areas of increased signal intensity on T2-weighted or FLAIR images without cavitation (signal distinct from CSF). Lesions limited to the subcortical gray matter or brainstem were excluded unless explicitly noted. When deep gray matter and brainstem hyperintensities were included, the collective term subcortical hyperintensities was used (**Figure S2**).

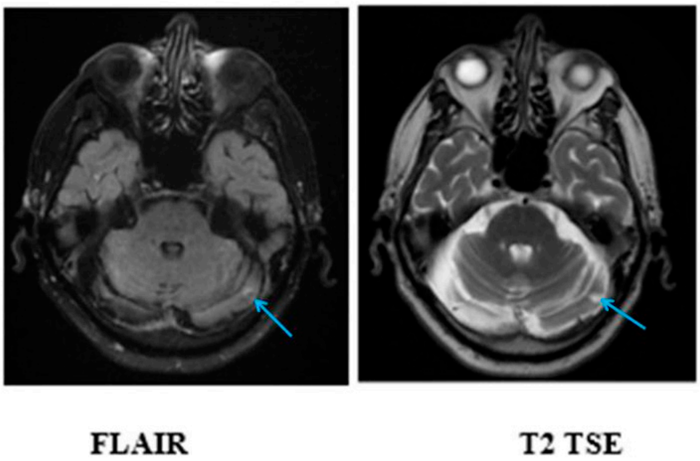

Figure S1. Lacune: Ovoid fluid-filled cavity (arrows).

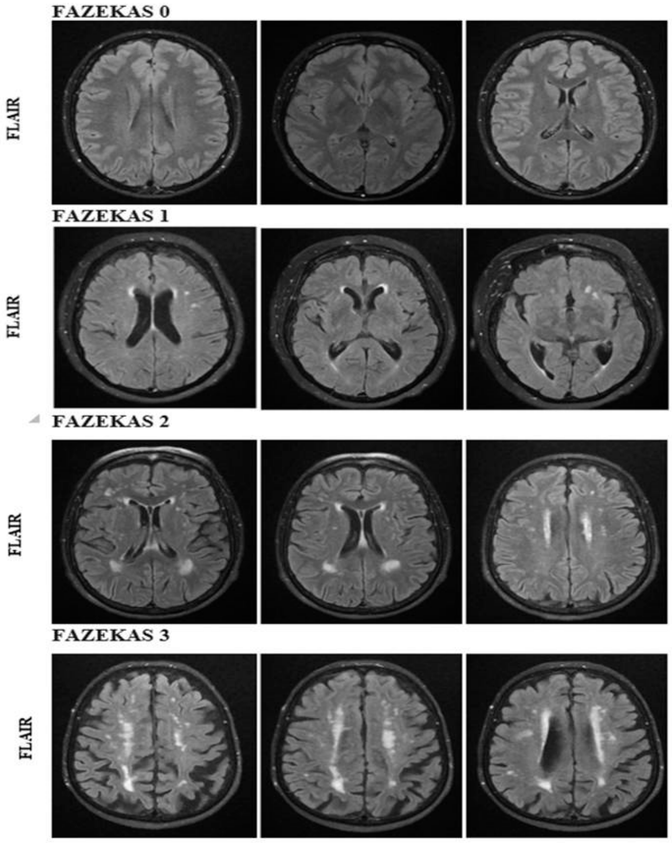

Figure S2. Variants of white matter changes: Fazekas 0: Normal; Fazekas 1: Multiple punctate lesions; Fazekas 2: Beginning confluences of lesions (bridging); Fazekas 3: Large confluent lesions.

(Section S4) Individual-level transport and minimal refit.

Locked phase: discrimination and calibration of the pre-specified model.

- Calibration intercept = 0.106; slope = 0.912
- Brier = 0.188
- AUC = 0.756

Table S2. Locked parameters used

Parameter	Value	Source
α_locked	-0.758	Baseline control log-odds used for the locked model
β_H_locked	1.293	log(OR_RE) from the literature meta-analysis
β_age_locked	0.022	Pre-specified age slope from controls-only model
β_E, β_A	0 (locked)	Neutral in locked phase

Note: This indicates strong external validity and acceptable patient-level prediction even without refitting. **α_locked = –0.758:** This intercept corresponds to the baseline log-odds of WMLs in PFO-negative controls, effectively setting the model's foundational risk level. It anchors the prediction space to the observed prevalence in the control (non-PFO) group, ensuring the model starts from a realistic local baseline. **β_age_locked = 0.022:** Estimated from a controls-only regression of WMLs on age, this coefficient captures the well-established contribution of aging to white matter injury, independent of PFO status. Locking this slope preserves the known, monotonic age effect across populations. **β_E and β_A = 0 (locked):** Neutral priors were assigned to the embolic (E-mode, including AF) and atrial/diastolic (A-mode) components, effectively turning these terms off during the initial locked-phase analysis. This allows pure testing of literature transportability for the shunt mechanism alone, before introducing AF or atrial remodeling influences in subsequent refitting.

Table S2 demonstrates the discipline and transparency of the model's design: all key priors were anchored to either external evidence (β_H_locked), biological constants (β_age_locked), or neutral assumptions (β_E, β_A). Such a locked structure provides a rigorous foundation for evaluating whether the shunt-related literature signal alone can explain WML risk in a real-world cohort—serving as a baseline against which the incremental contributions of AF (E-mode) and atrial cardiopathy (A-mode) can later be tested.

Table S3 summarizes the performance of the locked PAMAP model**,** which was applied without re-estimating parameters—thus serving as a strict test of transportability from the literature-trained specification to our clinical cohort.

Table S3. Model performance under locked priors

Metric	Value	Definition
Calibration intercept	0.106	GLM intercept with offset = logit(p_pred); ideal ≈ 0
Calibration slope	0.912	GLM coefficient on offset; ideal ≈ 1
Brier score	0.188	Mean squared error of probabilities
AUC	0.756	ROC area under curve

Notes: **Calibration intercept (0.106)** indicates that, on average, predicted probabilities were very close to observed event rates. An intercept near zero means the model did not systematically over- or underpredict the prevalence of white-matter lesions (WMLs). **Calibration slope (0.912)**, ideally equal to 1, suggests only mild underfitting. Predictions were slightly conservative, meaning the model slightly compressed the spread of individual risks but still maintained appropriate ranking across patients. **Brier score (0.188)**, a measure of overall prediction accuracy (with lower values indicating better performance), demonstrates **good probabilistic calibration** for a model transferred from literature priors to a real-world dataset. **AUC (0.756)** indicates **moderate-to-strong discrimination**, showing that the model correctly distinguished between patients with and without WMLs about 76% of the time—remarkably robust given that no local refitting was performed.

(Section S5) Minimal refit phase.

Table S4. Minimal Refit Coefficients (OR, 95 % CI)

**Variable**	**OR_adj**	**95 % CI**
Age	1.021	(1.007 – 1.036)
H (PFO)	2.452	(1.231 – 4.881)
E (AF)	1.318	(0.777 – 2.236)
A (Echo index)	1.187	(0.912 – 1.536)

Notes: **Age OR = 1.021 (95 % CI 1.007–1.036):** Each additional year of age was associated with a 2% higher odds of WMLs, consistent with the expected, monotonic age effect seen across cerebrovascular and neuroimaging studies. This reinforces age as a dominant background determinant of cerebral microvascular injury, independent of cardiac status. **H (PFO) OR = 2.452 (95 % CI 1.231–4.881):** The shunt/hypoxemia (H-mode) remained a robust and statistically significant predictor of WMLs even after minimal refit. Patients with TTE-detected PFO had approximately 2.5-fold higher odds of exhibiting moderate-to-severe WMLs compared with those without PFO. Clinically, this finding validates the literature-derived PFO–WML association under routine echocardiographic detection and supports the notion that shunt physiology—rather than undetected embolism or atrial arrhythmia—plays a key role in silent brain injury. **E (AF) OR = 1.318 (95 % CI 0.777–2.236):** The embolic/AF-related (E-mode) did not reach statistical significance. Although AF is a known cause of embolic stroke and cerebral microinfarction, in this TTE-defined cohort it did not independently explain WML burden once shunt-related and atrial-structural factors were accounted for. Clinically, this suggests that in patients with PFO and without established AF, silent WMLs are unlikely to be AF-driven, underscoring the mechanistic independence of PFO-related microvascular injury from classical cardioembolic pathways**. A (Echo index) OR = 1.187 (95 % CI 0.912–1.536):** The atrial/diastolic stress (A-mode) demonstrated a modest, non-significant trend toward higher WML risk with increasing left atrial and diastolic parameters (LAVi, LVMI, PASP). This may reflect early-stage atrial cardiomyopathy that coexists with PFO but does not yet exert a dominant cerebrovascular impact.

Table S5 summarizes model performance before and after the minimal refit. In this manuscript, the refit is not an intercept-only recalibration; it is a parsimonious re-estimation of the pre-specified Age, H, E, and A terms without adding new variables or interactions.

Table S5. Fit metrics before and after minimal refit

**Metric**	**Locked**	**Refit**	**Δ**
Brier	0.188	0.176	-0.012
AUC	0.756	0.783	+0.027

Note: **Brier Score (−0.012 improvement):** The Brier score, which measures the mean squared error between predicted and observed probabilities, improved modestly from 0.188 to 0.176 after minimal refit. Lower values indicate more accurate probabilistic predictions. This small but meaningful improvement shows that local calibration tuning helped the model's predicted probabilities better align with observed outcomes**,** enhancing reliability at the bedside—particularly when assessing individual patient risk for white-matter lesions (WMLs). **AUC (+0.027 improvement):** The area under the ROC curve (AUC) increased from 0.756 to 0.783, reflecting a notable gain in discrimination—the model's ability to correctly distinguish patients with and without WMLs. While the locked model already achieved good performance (AUC >0.75), the improvement following refit demonstrates that incorporating modest local adjustments (e.g., recalibrating the influence of atrial fibrillation and atrial indices) enhanced its clinical precision without overfitting**.**

(Section S6) Reproducibility resources and executable code.

The submission includes a de-identified patient-level specification table, a study-level literature extraction table, and a reviewer-ready code bundle.

The companion file "PAMAP_Code_Package.docx" contains two implementations: Code A (strict locked model with calibration-only update) and Code B (locked model with parsimonious Age/H/E/A refit). The current manuscript corresponds to Code B.

A short input schema and locked-coefficient template are included in the code bundle to facilitate replication and editorial review.

References:

1. Kossaify A, Nasr M. Diastolic Dysfunction and the New Recommendations for Echocardiographic Assessment of Left Ventricular Diastolic Function: Summary of Guidelines and Novelties in Diagnosis and Grading. *Journal of Diagnostic Medical Sonography*. 2019;35(4):317-325. doi:10.1177/8756479319836781
2. Zhang H, Tang H, Wu F, Yu C, Dong Q, Cao W. A score of non-contrast transthoracic echocardiography to screen patent foramen ovale in patients with embolic stroke of undetermined source. BMC Neurol. 2022 Feb 4;22(1):43. doi: 10.1186/s12883-022-02565-w. PMID: 35120481; PMCID: PMC8815249.
3. Ren P, Li K, Lu X, Xie M. Diagnostic value of transthoracic echocardiography for patent foramen ovale: a meta-analysis. Ultrasound Med Biol. 2013 Oct;39(10):1743-50. doi: 10.1016/j.ultrasmedbio.2013.03.016. Epub 2013 Jun 29. PMID: 23820251.
4. Fazekas F, Chawluk JB, Alavi A, Hurtig HI, Zimmerman RA. MR signal abnormalities at 1.5 T in Alzheimer's dementia and normal aging. AJR Am J Roentgenol. 1987 Aug;149(2):351-6. doi: 10.2214/ajr.149.2.351. PMID: 3496763.
