# Supplementary material for: Patent Foramen Ovale on Transthoracic Echocardiography and Brain White-Matter Hyperintensities: A Transportability Analysis and Practice-Anchored Risk Framework
Source: J Clin Med. 2026 Jun 11;15(12):4541. doi: 10.3390/jcm15124541 (PMC13301136; doi:10.3390/jcm15124541)
Supplement: Supplementary file 1 [file jcm-15-04541-s001.zip › jcm-4322739-supplementary/PAMAP Supplementary Code Package.pdf]

PAMAP Supplementary Code Package

Two manuscript-aligned code variants grouped in one file

Code A matches a calibration-only minimal refit. Code B matches a partial coefficient refit in which discrimination can change.

Required input files

- cohort.csv: patient_id, age, pfo_tte, af, a_mode_index, plus either wmh (0/1) or fazekas (0/1/2/3).
- locked_coefficients.csv: a two-column file named 'name' and 'value'.
- Required coefficient keys: alpha_locked, beta_age_locked, beta_H_locked. Optional keys: beta_E_locked, beta_A_locked.
- Outcome convention used here: if wmh is absent, then wmh is derived as 1{fazekas >= 1}.
- If there is an increase in AUC after refit, use Code B.

Code A — Strict locked model plus calibration-only update

Use this only for the minimal refit changes intercept and slope only.

```
#!/usr/bin/env python3
"""
S1 Code A. Strict locked model plus calibration-only update.

Use this version only if the manuscript states that the minimal refit
adjusts the intercept and slope only. Under this definition the AUC
should not change after refit, because the ranking of patients is
preserved.
"""

import argparse
import numpy as np
import pandas as pd


def sigmoid(x):
    return 1.0 / (1.0 + np.exp(-x))


def irls_intercept_slope(y, x, max_iter=100, tol=1e-8):
    """
    Fits: logit(p) = a + b * x
    using a two-parameter IRLS update.
    """
```

```
    y = np.asarray(y, dtype=float)
    x = np.asarray(x, dtype=float)
    X = np.column_stack([np.ones_like(x), x])
    beta = np.array([0.0, 1.0], dtype=float)

    for _ in range(max_iter):
        eta = X @ beta
        p = sigmoid(eta)
        w = np.clip(p * (1.0 - p), 1e-8, None)
        z = eta + (y - p) / w
        WX = X * w[:, None]
        lhs = WX.T @ X + 1e-10 * np.eye(2)
        rhs = WX.T @ z
        beta_new = np.linalg.solve(lhs, rhs)
        if np.max(np.abs(beta_new - beta)) < tol:
            beta = beta_new
            break
        beta = beta_new

    return float(beta[0]), float(beta[1])


def read_locked_coefficients(path):
    tab = pd.read_csv(path)
    coef = dict(zip(tab["name"].astype(str), tab["value"].astype(float)))

    required = ["alpha_locked", "beta_age_locked", "beta_H_locked"]
    missing = [k for k in required if k not in coef]
    if missing:
        raise ValueError(f"Missing locked coefficients: {missing}")

    coef.setdefault("beta_E_locked", 0.0)
    coef.setdefault("beta_A_locked", 0.0)
    return coef


def derive_outcome(df, fazekas_threshold=1):
    if "wmh" in df.columns:
        return pd.to_numeric(df["wmh"], errors="coerce")
    if "fazekas" in df.columns:
        faz = pd.to_numeric(df["fazekas"], errors="coerce")
        return (faz >= fazekas_threshold).astype(float)
    return None


def main():
    parser = argparse.ArgumentParser()
    parser.add_argument("--cohort_csv", required=True)
    parser.add_argument("--locked_csv", required=True)
    parser.add_argument("--fazekas_threshold", type=float, default=1.0)
    args = parser.parse_args()

    df = pd.read_csv(args.cohort_csv)
    coef = read_locked_coefficients(args.locked_csv)

    required_cols = ["patient_id", "age", "pfo_tte", "af", "a_mode_index"]
    missing = [c for c in required_cols if c not in df.columns]
```

```
    if missing:
        raise ValueError(f"Missing cohort columns: {missing}")

    age = pd.to_numeric(df["age"], errors="coerce")
    pfo = pd.to_numeric(df["pfo_tte"], errors="coerce").fillna(0.0)
    af = pd.to_numeric(df["af"], errors="coerce").fillna(0.0)
    a_mode = pd.to_numeric(df["a_mode_index"], errors="coerce").fillna(0.0)

    eta_locked = (
        coef["alpha_locked"]
        + coef["beta_age_locked"] * age
        + coef["beta_H_locked"] * pfo
        + coef["beta_E_locked"] * af
        + coef["beta_A_locked"] * a_mode
    )
    p_locked = sigmoid(eta_locked)

    pd.DataFrame(
        {
            "patient_id": df["patient_id"],
            "eta_locked": eta_locked,
            "p_locked": p_locked,
        }
    ).to_csv("pred_locked.csv", index=False)

    y = derive_outcome(df, fazekas_threshold=args.fazekas_threshold)
    if y is not None and y.notna().all():
        a_hat, b_hat = irls_intercept_slope(y.to_numpy(), eta_locked.to_numpy())
        p_calibrated = sigmoid(a_hat + b_hat * eta_locked.to_numpy())

        pd.DataFrame(
            {
                "patient_id": df["patient_id"],
                "p_calibrated": p_calibrated,
            }
        ).to_csv("pred_calibrated.csv", index=False)

        with open("calibration_params.txt", "w", encoding="utf-8") as f:
            f.write(f"a_hat={a_hat:.6f}\n")
            f.write(f"b_hat={b_hat:.6f}\n")

        print("Wrote pred_locked.csv, pred_calibrated.csv, calibration_params.txt")
    else:
        print("Wrote pred_locked.csv; no complete outcome column was found.")


if __name__ == "__main__":
    main()
```

Code B — Locked model plus partial coefficient refit

Use this only when E and A are unlocked, or if the post-refit AUC changes.

```
#!/usr/bin/env python3
"""
S2 Code B. Locked model plus partial coefficient refit.

Use this version only if the manuscript states that E and A were
unlocked during refit, or if the manuscript reports a changed AUC after
refit. A changed AUC implies that patient ranking changed, which is not
a calibration-only update.
"""

import argparse
import math
import numpy as np
import pandas as pd


def sigmoid(x):
    return 1.0 / (1.0 + np.exp(-x))


def irls_logistic(X, y, max_iter=200, tol=1e-8):
    """
    Generic logistic regression by IRLS.
    """
    X = np.asarray(X, dtype=float)
    y = np.asarray(y, dtype=float)
    beta = np.zeros(X.shape[1], dtype=float)

    for _ in range(max_iter):
        eta = X @ beta
        p = sigmoid(eta)
        w = np.clip(p * (1.0 - p), 1e-8, None)
        z = eta + (y - p) / w
        WX = X * w[:, None]
        lhs = WX.T @ X + 1e-8 * np.eye(X.shape[1])
        rhs = WX.T @ z
        beta_new = np.linalg.solve(lhs, rhs)
        if np.max(np.abs(beta_new - beta)) < tol:
            beta = beta_new
            break
        beta = beta_new

    return beta


def auc_rank(y, score):
    """
    Rank-based AUC. Assumes y is binary 0/1.
    """
    y = np.asarray(y, dtype=int)
    score = np.asarray(score, dtype=float)

    order = np.argsort(score)
```

```
    ranks = np.empty_like(order, dtype=float)
    ranks[order] = np.arange(1, len(score) + 1, dtype=float)

    n1 = y.sum()
    n0 = len(y) - n1
    if n1 == 0 or n0 == 0:
        return np.nan

    rank_sum_pos = ranks[y == 1].sum()
    return (rank_sum_pos - n1 * (n1 + 1) / 2.0) / (n1 * n0)


def brier_score(y, p):
    y = np.asarray(y, dtype=float)
    p = np.asarray(p, dtype=float)
    return float(np.mean((y - p) ** 2))


def read_locked_coefficients(path):
    tab = pd.read_csv(path)
    coef = dict(zip(tab["name"].astype(str), tab["value"].astype(float)))
    required = ["alpha_locked", "beta_age_locked", "beta_H_locked"]
    missing = [k for k in required if k not in coef]
    if missing:
        raise ValueError(f"Missing locked coefficients: {missing}")
    coef.setdefault("beta_E_locked", 0.0)
    coef.setdefault("beta_A_locked", 0.0)
    return coef


def derive_outcome(df, fazekas_threshold=1):
    if "wmh" in df.columns:
        return pd.to_numeric(df["wmh"], errors="coerce")
    if "fazekas" in df.columns:
        faz = pd.to_numeric(df["fazekas"], errors="coerce")
        return (faz >= fazekas_threshold).astype(float)
    raise ValueError("Outcome not found. Provide 'wmh' or 'fazekas'.")


def main():
    parser = argparse.ArgumentParser()
    parser.add_argument("--cohort_csv", required=True)
    parser.add_argument("--locked_csv", required=True)
    parser.add_argument("--fazekas_threshold", type=float, default=1.0)
    args = parser.parse_args()

    df = pd.read_csv(args.cohort_csv)
    coef = read_locked_coefficients(args.locked_csv)

    required_cols = ["patient_id", "age", "pfo_tte", "af", "a_mode_index"]
    missing = [c for c in required_cols if c not in df.columns]
    if missing:
        raise ValueError(f"Missing cohort columns: {missing}")

    age = pd.to_numeric(df["age"], errors="coerce")
    pfo = pd.to_numeric(df["pfo_tte"], errors="coerce").fillna(0.0)
    af = pd.to_numeric(df["af"], errors="coerce").fillna(0.0)
```

```
a_mode = pd.to_numeric(df["a_mode_index"], errors="coerce").fillna(0.0)
y = derive_outcome(df, fazekas_threshold=args.fazekas_threshold)

keep = age.notna() & pfo.notna() & af.notna() & a_mode.notna() & y.notna()
df = df.loc[keep].copy()
age = age.loc[keep].to_numpy()
pfo = pfo.loc[keep].to_numpy()
af = af.loc[keep].to_numpy()
a_mode = a_mode.loc[keep].to_numpy()
y = y.loc[keep].to_numpy()

eta_locked = (
    coef["alpha_locked"]
    + coef["beta_age_locked"] * age
    + coef["beta_H_locked"] * pfo
    + coef["beta_E_locked"] * af
    + coef["beta_A_locked"] * a_mode
)
p_locked = sigmoid(eta_locked)

X_refit = np.column_stack(
    [
        np.ones(len(df)),
        age,
        pfo,
        af,
        a_mode,
    ]
)
beta = irls_logistic(X_refit, y)
eta_refit = X_refit @ beta
p_refit = sigmoid(eta_refit)

refit_table = pd.DataFrame(
    {
        "parameter": [
            "alpha_refit",
            "beta_age_refit",
            "beta_H_refit",
            "beta_E_refit",
            "beta_A_refit",
        ],
        "log_odds": beta,
        "odds_ratio": np.exp(beta),
    }
)
refit_table.to_csv("refit_coefficients.csv", index=False)

pd.DataFrame(
    {
        "patient_id": df["patient_id"],
        "p_locked": p_locked,
        "p_refit": p_refit,
    }
).to_csv("pred_refit.csv", index=False)

with open("fit_metrics.txt", "w", encoding="utf-8") as f:
```

```
        f.write(f"locked_brier={brier_score(y, p_locked):.6f}\n")
        f.write(f"locked_auc={auc_rank(y, p_locked):.6f}\n")
        f.write(f"refit_brier={brier_score(y, p_refit):.6f}\n")
        f.write(f"refit_auc={auc_rank(y, p_refit):.6f}\n")

    print("Wrote refit_coefficients.csv, pred_refit.csv, fit_metrics.txt")


if __name__ == "__main__":
    main()
```
